# Identification of an Immune signature assisted prognosis, and immunotherapy prediction for IDH wildtype glioblastoma

**DOI:** 10.7150/jca.100144

**Published:** 2024-10-21

**Authors:** Xuetao Han, Huandi Zhou, Xiaohui Ge, Liubing Hou, Haonan Li, Dongdong Zhang, Yu Wang, Xiaoying Xue

**Affiliations:** 1 Department of Radiotherapy, The Second Hospital of Hebei Medical University, Shijiazhuang 050000, Hebei Province, China.; 2 Department of Central Laboratory, The Second Hospital of Hebei Medical University, Shijiazhuang 050000, Hebei Province, China.

**Keywords:** Glioblastoma, FMOD, MXRA5, RAB36, immune cell infiltration, immunotherapy

## Abstract

IDH-wildtype glioblastoma (GBM) is the most common and malignant primary brain tumor. The purpose of this study is to establish a prognostic gene signature for IDH-wildtype GBM. RNA sequencing data of normal brain tissue and GBM patients were obtained from TCGA, CGGA, GEO and the GTEx databases. Identification of prognostic differentially expressed genes (DEGs) with | log2 fold change | > 0.5 and adjust p < 0.05 in TCGA and CGGA databases by "limma" method. By LASSO regression analysis and multivariate Cox analysis, a 3-gene prognostic signature composed of FMOD, MXRA5 and RAB36 was established. The 3-gene prognostic risk model is validated by TCGA and GSE43378 datasets. The expression of FMOD, MXRA5 and RAB36 in GBM patients was significantly higher than that in normal brain tissues in CCGA, TCGA and GSE29796 data sets. In order to further verify this result, total RNA was extracted from tumors and paracancerous tissues of 9 GBM patients. RT-PCR results showed that the expression of FMOD, MXRA5 and RAB36 in tumor tissues of most patients was higher than that in paracancerous tissues. The results of GSEA showed that the pathway enrichment of the 3-gene signature was mainly related to tumor immunity. Immune cell infiltration analyzed by ssGSEA showed that there were significant differences in macrophages between high- and low-risk groups. Immune checkpoint genes correlation analysis showed that PD-L1 gene expression is closely related to risk score. Our study identifies a prognostic-associated risk model and provides a potential effective immunotherapy target for IDH-wildtype GBM patients.

## Introduction

World Health Organization (WHO) grade 4 gliomas are the most common primary malignant intracranial tumors in adults^1^. In the 2021 WHO Classification of Tumors of the Central Nervous System, gliomas were further classified on the basis of histological and molecular features^2^. WHO grade 4 gliomas are classified according to IDH gene status into IDH-mutant astrocytomas, grade 4, and IDH-wildtype glioblastoma (GBM)^2^. The prognosis of IDH-wildtype GBM is poor, despite standard treatment, the median survival of patients with IDH wild-type GBM is still less than 14 months^3^. Emerging biological studies has to some extent improved diagnostic and therapeutic strategies for IDH-wildtype GBM; however, there has been no breakthrough, owing to both tumor heterogeneity and a limited understanding of pathogenesis of this type of GBM. Therefore, there is a need to further elucidate the pathogenesis of IDH-wildtype GBM and identify new biomarkers for prediction of prognosis and therapeutic effect.

The progression of IDH-wildtype GBM requires not only genetic driving factors but also microenvironmental interactions^4^. The tumor microenvironment (TME), which has an important role in tumor growth, is mainly composed of tumor cells and fibroblasts, immune and inflammatory cells, glial cells, microvessels, and infiltrating biomolecules^5^. Tumor cells interact with their microenvironment to promote tumorigenesis. Many methods are available for estimation of tumor cell type and fraction using RNA sequencing data. These provide a landscape of the TME that can be used to study mechanisms of tumor progression and identify potential new immunotherapies. Glioma-associated microglia macrophages (GAM) are the most functional cells in the glioma TME, accounting for 30-50% of total cells^6^. Monocyte-derived macrophages are more abundant in IDH-wildtype GBM and recurrent tumors, and microglia represent the main population in IDH mutant gliomas^4^. Under the influence of different stimulating factors, macrophages can polarize into either pro-inflammatory/anti-tumor or anti-inflammatory/pro-tumorigenic phenotypes; that is, they have phenotypic plasticity^7^. Macrophages also exhibit heterogeneity, and different macrophage subpopulations have different effects on tumor occurrence and progression. At the initial stage of tumor development, macrophages may mainly have a pro-inflammatory role and inhibit tumor development^8^ . As tumor development progresses, macrophages in the TME develop M2-like phenotypes owing to the action of various stimulating factors. These cytotoxic macrophages are transformed into tumor-supporting macrophages, further promoting tumor progression^9^. In glioma, GAM are more likely to have an anti-inflammatory and pro-tumor phenotypes, enabling them to enhance glioma invasion, angiogenesis, and tumor growth and contribute to the immunosuppressive TME^10^. Thus, the effects of the TME are inseparable from those of tumor cells. Furthermore, the heterogeneity of TME may result in different response rates of tumor patients to immunotherapy^11^.

Several large sequencing databases for glioma have been established; these can be used to better understand the mechanisms of glioma transformation and progression and to provide new ideas for prognostic prediction and treatment. In addition, the advent of single-cell sequencing has helped to lay a foundation for study of the genetic features of tumor subclones and has deepened our understanding of the characteristics of immune cell infiltration and the immune microenvironment. In this study, the Cancer Genome Atlas (TCGA), the Genotype-Tissue Expression Project (GTEx), Gene Expression Omnibus (GEO) and Chinese Glioma Genome Atlas (CGGA) databases were used to establish a prognostic risk model to accurately and effectively predict prognosis. In addition, the immune cell infiltration and immune microenvironment of IDH-wildtype GBM were further analyzed to evaluate potential approaches to immunotherapy.

## Material and Methods

### Data acquisition and processing

**Figure 1:**
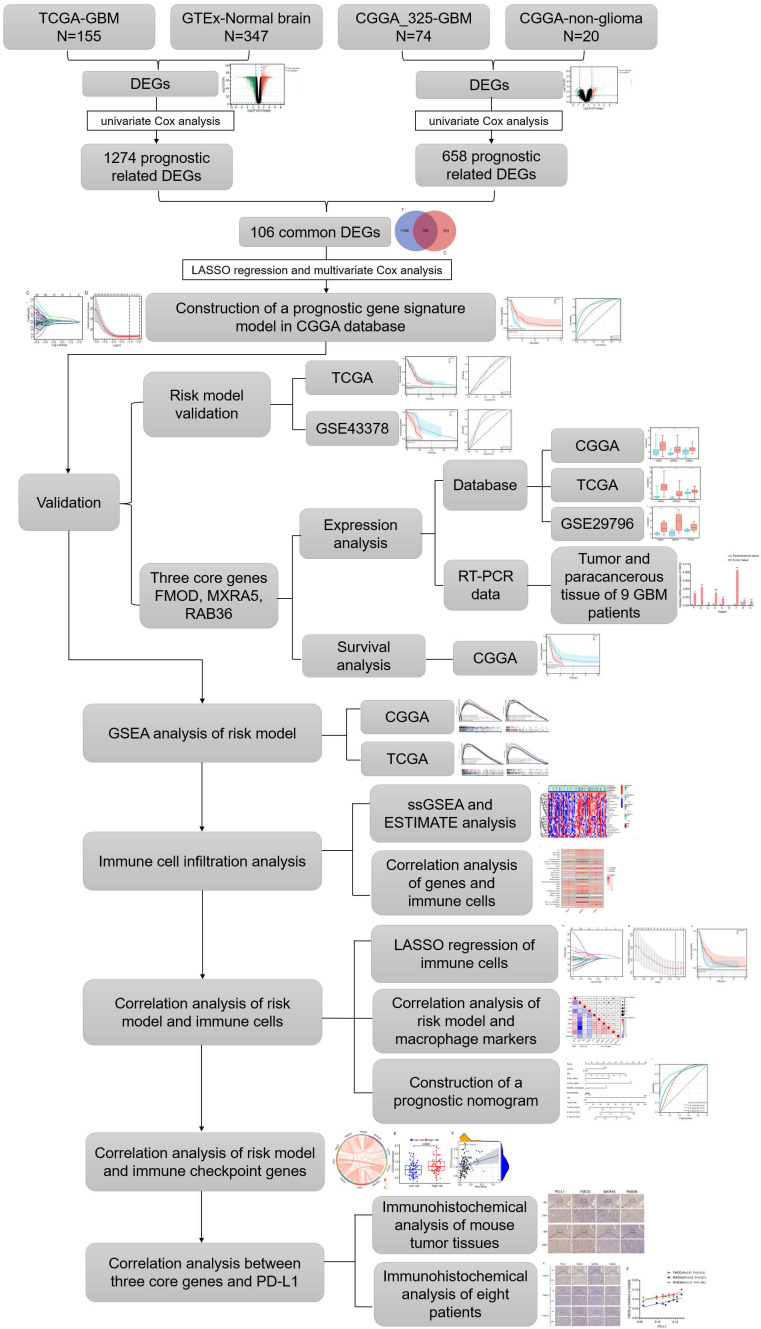
** Flow chart of study design.** Abbreviations: CGGA (Chinese Glioma Genome Atlas). TCGA (The Cancer Genome Atlas). GTEx (The Genotype-Tissue Expression). DEGs (differentially expressed genes). GSEA (Gene Set Enrichment Analysis). ESTIMATE (Estimation of STromal and Immune cells in MAlignant Tumours using Expression data). LASSO (The Least Absolute Shrinkage and Selection Operator).

RNA sequencing data from GBM patients were obtained from TCGA (https://portal.gdc.cancer.gov/), CGGA (http://www.cgga.org.cn, mRNAseq 325 dataset) and GEO (http://www.ncbi.nlm.nih.gov/geo/, GSE43378, GSE29796) database. The sequencing data of normal brain tissues were obtained from the GTEx (http://commonfund.nih.gov/GTEx/) and CGGA database (20 samples for non-glioma as control dataset). To eliminate the batching effect between the GTEx and TCGA datasets, we used the “limma” package of R software to integrate the two datasets. Similarly, CGGA_325 dataset and non-glioma as control dataset were normalized by “limma” package. According to the 2021 WHO classification of the central nervous system tumors, we selected the sequencing data of WHO grade 4 gliomas for analysis. The baseline data of glioma patients in this study can be found in Supplementary Table 1, and detailed information such as patient ID can be found in Supplementary File 1. The flowchart is shown in Figure 1.

### Differentially expressed genes (DEGs)

“Limma” package was used to analyze the DEGs between TCGA data and GTEx data. The DEGs in CGGA_325 dataset and non-glioma as control dataset were analyzed by the same method. The cutoff criteria were set as | log2 fold change (FC) | > 0.5 and adjust p < 0.05. We screened out prognostic related genes by univariate Cox analysis of DEGs. Then the prognostic related DEGs of TCGA and CGGA are intersected.

### Construction of a prognostic risk score model

Clinical information from CGGA_325 dataset was used for further analysis. The least absolute shrinkage and selection operator (LASSO) method and multivariate Cox regression were used to form the final risk score model. Gene coefficient is the beta value in multivariate Cox analysis. The risk score for each patient was calculated using the following formula (i represents the target prognostic related DEG): Risk score = ∑ coef (i)*log 2 (counts (i)+1). Patients were divided into high-and low-risk groups based on the median value of risk score. The predictive ability of the risk score model was evaluated by Kaplan-Meier survival analysis, univariate Cox regression analysis and multivariate Cox regression analysis.

### Validation of the risk score model

TCGA database and GSE43378 dataset are used to validate the risk score model. The risk score of each patient was calculated using the risk score formula. Patients were divided into high- and low-risk group according to the median risk score. Kaplan-Meier survival analysis and receiver operating characteristic (ROC) curve were used to evaluate and verify the risk scoring model.

### Functional enrichment analysis

The Gene Ontology (GO) and Kyoto Encyclopedia of Genes and Genomes (KEGG) enrichment analysis of high- and low-risk groups were performed by Gene Set Enrichment Analysis (GSEA) software (c2.cp.kegg.v7.5.1.symbols and c5.go.v7.5.1.symbols). A normalized enrichment score (NES) >1 and FDR <0.05 were considered meaningful.

### Immune microenvironment analysis

Estimation of STromal and Immune cells in MAlignant Tumours using Expression data (ESTIMATE) and single-sample gene-set enrichment analysis (ssGSEA) were used to evaluate the tumor immune microenvironment. The ssGSEA is performed by “gsva” package containing 29 immune infiltration-related information. Then analyze the difference of immune microenvironment and immune cell infiltration between high- and low-risk groups. Univariate Cox regression was used to analyze the relationship between immune cells and the prognosis of GBM patients, and to find the prognosis-related immune cells. LASSO method and multivariate Cox regression were used to determine the main immune cells related to prognosis. We established a nomogram to integrate risk scores, prognostic immune cells, and clinical features to improve the accuracy of prognostic prediction.

### RNA extraction and Quantitative real-time PCR

Nine postoperative fresh frozen tissue samples of GBM patients were collected from the Neurosurgery Department of the Second Hospital of Hebei Medical University from May 2019 to December 2019, including tumor and paracancerous tissue. Paracancerous tissue is defined as 2cm outside the tumor edge identified by multimodal neuronavigation before operation. This study was reviewed and approved by the Ethics Committee of Second Hospital of Hebei Medical University (approval number: 2019-R191). In this study, informed consent was obtained from all patients by completing an informed consent form. The total RNA of tissue was extracted by TriZol reagent (Invitrogen, USA). The obtained RNA was reverse transcribed into cDNA by Hifair® Ⅲ 1st Strand cDNA Synthesis SuperMix for qPCR (Yeasen, China). Real-time PCR was performed based on Hieff UNICON® Universal Blue qPCR SYBR Green Master Mix (Yeasen, China). The primer sequences of the three genes are as follows: 5′-TCCAAGAGGATGATCGACGC-3′ (forward) and 5′-TGTGTTCAATCTTGGCCGGT-3′ (reverse) for MXRA5; 5′-ACCGTCCCCGATAGCTACTT-3′ (forward) and 5′-CATCCTGGACCTTCCAGCAAA-3′ (reverse) for FMOD; 5′-ATCGAGACTACAAGGCCAC-3′ (forward) and 5′-CTGTGTCCCAGATCTGGAG-3′ (reverse) for RAB36. As an endogenous control, the sequence of GAPDH primers is 5′-CATGAGAAGTATGACAACAGCCT-3′ (forward) and 5′-AGTCCTTCCACGATACCAAAGT-3′ (reverse). Forty cycles of PCR were carried out in the Real-Time PCR Detection System (Bio-Rad, USA) for amplification. Relative expression levels were determined by 2 -ΔΔCT, which was calculated by subtracting the CT value of GAPDH from the CT value of three genes.

### Immunohistochemical (IHC) staining

The tissue wax blocks diagnosed as gliomas in the Department of Pathology of the second Hospital of Hebei Medical University were collected and sliced and stained with immunohistochemical kit (PV-9000, ZSGB-BIO, China). The specific steps are as follows: first, 3% peroxidase solution is used for antigen repair to eliminate endogenous enzymes in the tissue. Then, the first antibody will be incubated overnight. The next day, wash the tissue three times with phosphate buffered saline (PBS). After incubating in reaction enhancement solution, the tissue and the second antibody were incubated at room temperature for 30 minutes. After washing with PBS for three times, the tissue was stained with DAB staining solution, then re-stained with hematoxylin, ammonia-resistant blue, alcohol dehydration, and sealed with neutral glue. Primary antibodies (FMOD, MXRA5, and PD-L1) for IHC were purchased from Proteintech (Rosemont, IL, USA). The catalog numbers and recommended working concentrations of the antibodies are as follows: FMOD: Cat No. 60108-1-Ig, dilution 1:200; MXRA5: Cat No. 25472-1-AP, dilution 1:200; PD-L1: Cat No. 66248-1-Ig, dilution 1:1000. The primary antibody of RAB36 was purchased from Bioss company (Beijing, China), Cat No. bs-21084R, with a dilution of 1:200. The immunohistochemical sections were scanned in a digital pathological panoramic scanner (KFMI, Ningbo, China), and 5 visual fields of 200× were randomly selected, and the average optical density was calculated by ImageJ software. GraphPad Prism 8 software was used for correlation analysis.

### Cell culture

In this study, the mouse glioma cell lines GL261-*luci* and CT2A-*luci* were donated by the Department of Neurology Laboratory of the second Hospital of Hebei Medical University. All cells were cultured in Dulbecco's modified eagle medium (DMEM) supplemented with 10 % fetal bovine serum and 1 % penicillin/streptomycin with a 37 °C moist environment containing 5 % CO2.

### Establishment of mouse model

Male C57BL/6N mice at 8 weeks of age were purchased from Beijing Vital River Laboratory Animal Technology (Beijing, China). For the glioma model, mouse glioma CT2A or GL261 cells were suspended in PBS to achieve a final cell density of 1×10^7^/mL (5×10^6^ cells in 500μL PBS). Each mouse was injected with 100μL of cell suspension, with 5 mice in each group. The injection site was in the subcutaneous tissue of the left lower limb of the mouse. After the tumor formed, the mice were imaged *in vivo*. When the size of the tumor was about 1cm^3^, all mice were killed by cervical dislocation and the tumor tissue was removed. After the tumor tissue was fixed and embedded, the protein expression of FMOD, RAB36, MXRA5, and PD-L1 was detected by IHC assay. The animal protocol has been approved by the Ethics Committee of the Second Hospital of Hebei Medical University (approval number: 2023-AE329).

### *In vivo* imaging of mice

Each mouse was intraperitoneally injected with 200μL D-fluorescein potassium salt (15mg/mL, Yeasen, China) and some of the hair around the tumor was shaved off. Then place the mice in an inhalation anesthesia induction box containing 2% isoflurane to completely anesthetize them. After approximately 10 min, the bioluminescence was observed using IVIS Spectrum (PerkinElmer, USA).

### Statistical analysis

R version 4.1.2 (Statistics Department of the University of Auckland) and the corresponding packages was applied for correlation analysis. Graphpad Prism, version 8 (GraphPad Software, San Diego, California USA) was used for statistical analyses. The normal (Gaussian) distributions of data were evaluated by using the Shapiro-Wilk test, and then the Mann-Whitney test, Dunn's test, or the Kruskal-Wallis test was conducted to compare differences between the groups. Kaplan-Meier curve analysis was used to evaluate the survival difference between the high- and low-risk groups. Pearson correlation analysis was used for correlation analysis. P < 0.05 was considered statistically significant.

## Results

### Identification of prognostic related DEGs in GBM

**Figure 2:**
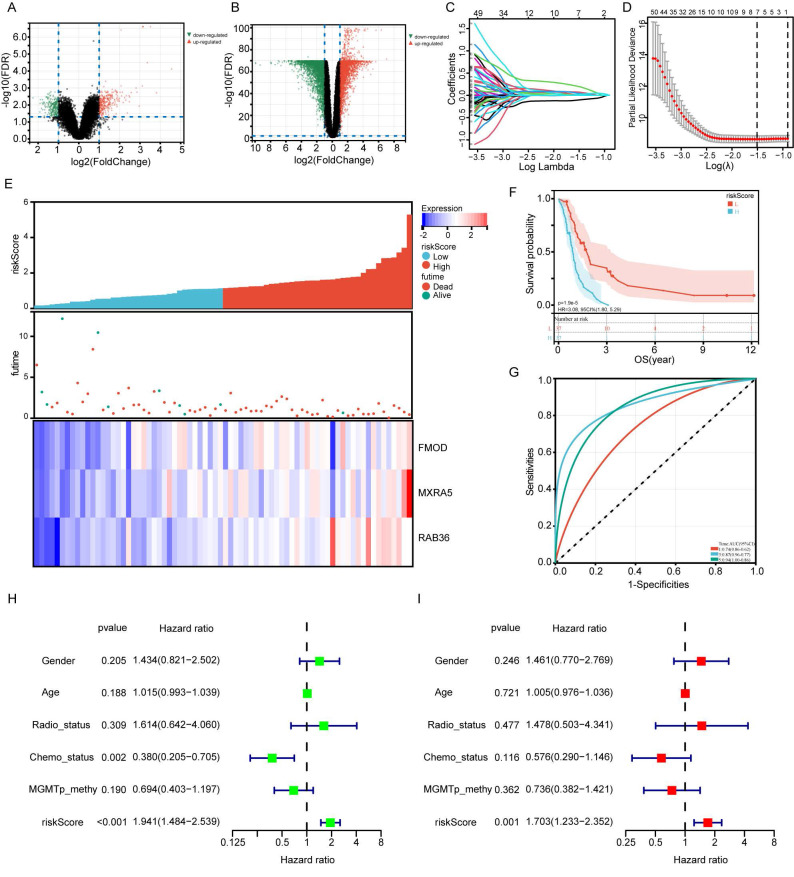
** Construction of a prognostic risk signature by CGGA database.** A: The volcano plot of differentially expressed genes (DEGs) in CGGA_325 dataset and non-glioma as control dataset. B: The volcano plot of DEGs in TCGA data and GTEx data. C: Cross-validation for tuning the coefficient selection in the least absolute shrinkage and selection operator (LASSO) regression. D: LASSO regression of the 106 prognostic related DEGs. E: Allocation of patients in CGGA_325 database on the basis of the risk score. F: Kaplan-Meier curves display the diversity in OS between the high-risk and low-risk groups in CGGA_325 database. G: Area Under the Curve (AUC) of time-dependent receiver operating characteristic (ROC) curves examined the prognostic performance of the risk score. H-I: Univariate (H) and multivariate (I) Cox regression analyses of the association between clinic pathological factors and OS of patients.

The flowchart of the study is summarized in Figure 1. We combined and standardized the sequencing data of primary GBM patients in TCGA database (155 samples) and normal brain tissue data in GTEx database (347 samples). 12444 DEGs were identified between normal and GBM tissues in TCGA, of which 6146 were upregulated and 6298 downregulated (Figure 2A). 1274 prognostic related DEGs were identified by univariate Cox analysis. The sequencing data of primary GBM patients in CGGA_325 dataset and non-glioma as control dataset were also combined and standardized. The 2122 DEGs with 1249 upregulated and 873 downregulated were identified in CGGA databases (Figure 2B). 657 prognostic related DEGs were identified by univariate Cox analysis. There were 106 common genes in two different prognostic-related DEGs sets (Supplementary Figure 1A). The prognostic related DEGs in CGGA and TCGA databases can be found in Supplementary File 2.

### Construction of a prognostic gene signature model

LASSO regression was used to analyze the dimensionality reduction of 106 prognostic DEGs (Figure 2C-D). The LASSO regression analysis results show that the optimal λ value at which the deviation is minimized is -1.5. At this point, the model achieves the highest fitting performance, and a total of 7 variables were selected: CPB2-AS1, DNAH9, FMOD, MXRA5, RAB36, RCN1, TAGLN2. Then multivariate Cox regression was used to establish a prognostic model composed of fibromodulin (FMOD), matrix-remodeling associated protein 5 (MXRA5) and RAB36. The risk score for each sample was calculated according to the expression levels and coefficient values of the three genes. Risk score = (0.159*FMOD_exp_) + (0.228*MXRA5_exp_) + (0.684*RAB36_exp_). Patients in the CGGA dataset were stratified according to their risk scores (Figure 2E). Kaplan-Meier survival curve showed that patients in the high-risk group had significantly poorer prognosis compared with those in the low-risk group (Figure 2F, P<0.001). In addition, ROC curve analysis also showed that the established model could well predict the prognosis of GBM patients (Figure 2G, 1-year, 3-year and 5-year ROC area under the curve (AUC) were 0.74, 0.87 and 0.94, respectively). The Brier score curve was showed in Supplementary Figure 1B. Risk score was statistically associated with overall survival (OS) according to univariate Cox regression analysis (Figure 2H, HR=1.941, P<0.001). Multivariate analysis confirmed thatit remained an independent prognostic index for OS in patients with IDH1-wildtype GBM (Figure 2I, HR=1.703, P=0.001). These results suggest that the risk score has potential clinical applications in prognostic evaluation of these patients.

### Validation of prognostic gene signature model

**Figure 3:**
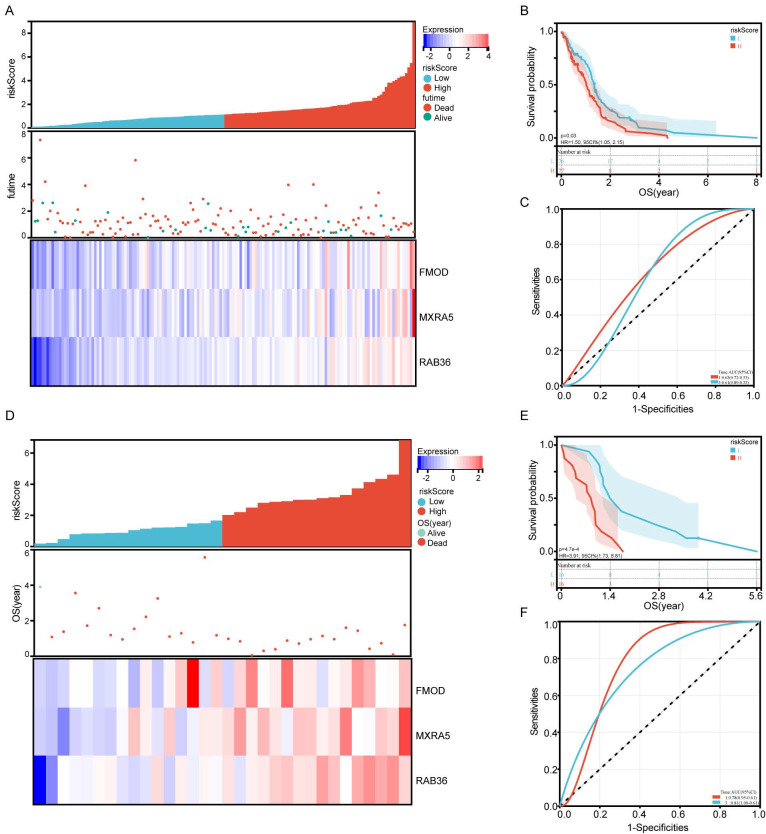
** Validation of the prognostic risk signature by TCGA database and GSE43378 dataset.** A: Allocation of patients in TCGA database on the basis of the risk score. B: Kaplan-Meier curves display the diversity in OS between the high-risk and low-risk groups in TCGA database. C: AUC of time-dependent ROC curves of the risk score in TCGA. D: Allocation of patients in GSE43378 dataset on the basis of the risk score. E: Kaplan-Meier curves display the diversity in OS between the high-risk and low-risk groups in GSE43378 dataset. F: AUC of time-dependent ROC curves of the risk score in GSE43378 dataset.

TCGA and GSE43378 datasets were used for validation of the risk model. Patients were divided into high-risk and low-risk groups based on the median risk score (Figure 3A and D). Patients in the high-risk group had significantly lower OS than those in the low-risk group both in TCGA and GSE43378 datasets (Figure 3B and E, P=0.03, P<0.001, respectively). As shown in Figure 3C and F, the AUC reached 0.63 at 1 year, 0.61 at 3 years in TCGA database, and 0.78 at 1 year, 0.81 at 3 years in GSE43378 datasets. The Brier score curve of TCGA and GSE43378 were showed in Supplementary Figure 1C-D.

**Figure 4:**
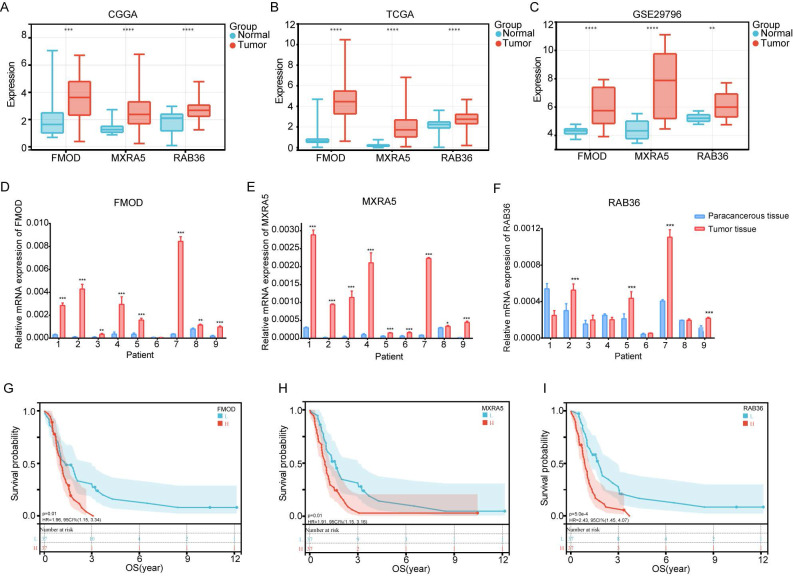
** Expression, survival and correlation analysis of FMOD, MXRA5 and RAB36.** A-C: Gene expression difference between normal brain tissue and GBM tissue in CGGA (A), TCGA (B) and GSE29796 (C). D-F: Gene expression difference between paracancerous tissue and tumor tissue from 9 GBM paitents. G-I: Survival analysis of FMOD (G), MXRA5 (H) and RAB36 (I) according to the comparison of high and low groups of gene expression.

We further analyzed the expression differences of the three genes between normal brain tissues and tumor tissues in the CGGA, TCGA and GSE29796 datasets. The results were highly consistent across the three databases, showing that the expression of these genes in GBM tumor tissue was higher than that in normal brain tissue (Figure 4A-C). We determined gene expression in nine cancerous and tumor- adjacent tissues from patients with GBM and found that expression levels of FMOD, MXRA5, and RAB36 in the cancer tissue was higher than those in the para-cancerous tissue in most patients (Figure 4D-F). Thus, these results were consistent with those of the database analysis. Analysis of the CGGA data showed that the survival of patients with high expression of these three genes was lower than that of patients with low expression (Figure 4G-I). The basic information of the nine patients is provided in Supplementary File 1.

### Gene Set Enrichment Analysis of risk model

**Figure 5:**
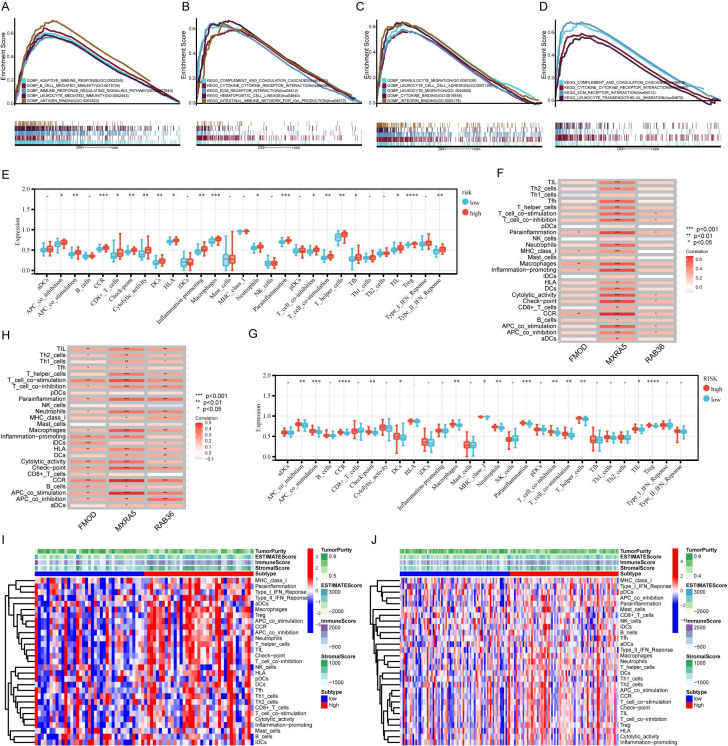
** Gene set enrichment analysis (GSEA) and correlation analysis of immune factors.** A-B: GSEA results based on the GO (A) and KEGG (B) gene sets of CGGA database. C-D: GSEA results based on the GO (C) and KEGG (D) gene sets of TCGA database. E: The distribution of tumor-infiltrating immune cells based on the ssGSEA algorithm in CGGA database. F: The correlations between 3 prognostic related genes and immune cells in CGGA database. G: The distribution of tumor-infiltrating immune cells based on the ssGSEA algorithm in TCGA database. H: The correlations between 3 prognostic related genes and immune cells in TCGA database. I-J: The landscapes of high- and low-risk groups based on ssGSEA and ESTIMATE analysis in CGGA (I) and TCGA (J) databases.

Functional enrichment was analyzed for the high- and low-risk groups by GSEA. In CGGA database, GO term analysis revealed that high-risk group was significantly enriched in the biological processes (BP) of adaptive immune response, B cell mediated immunity, immune response regulating signaling pathway, leukocyte mediated immunity and cellular components (CC) of antigen binding (Figure 5A). KEGG analysis revealed that the high-risk group was linked to complement and coagulation cascades, cytokine-cytokine receptor interaction, ECM receptor interaction, hematopoietic cell lineage, and intestinal immune network for IgA production (Figure 5B). We also performed enrichment analysis for the high-and low-risk groups based on TCGA data. The high-risk group showed significant enriched in the BP of granulocyte migration, leukocyte cell-cell adhesion, leukocyte migration, and molecular functions (MF) of cytokine binding, integrin binding (Figure 5C). KEGG analysis revealed that high-risk group was enriched in complement and coagulation cascades, cytokine-cytokine receptor interaction, ECM receptor interaction, and leukocyte transendothelial migration (Figure 5D). GO and KEGG analyses showed that the high-and low-risk groups were strongly associated with immunity according to both CGGA and TCGA data. These results suggest that the three-gene signature used to construct our prognostic risk model is related to tumor immunity.

### Immune cell infiltration analysis

The composition of immune cells in the TME was evaluated using ssGSEA algorithm. According to the CGGA data, the infiltration frequency of B cells, CD8+T cells, dendritic cells, macrophages, neutrophils and regulatory T (Treg) cells was higher in the high-risk group than that in the low-risk group (Figure 5E). According to TCGA data, infiltration of macrophages, neutrophils, and Treg cells was higher in the high-risk group (Figure 5G). We further analyzed the correlations of each of the three genes with immunity and found that all three were related to immunity, especially MXRA5 (Figure 5F and H). The results of ESTIMATE analysis showed that immune scores and stromal scores were higher in the high-risk group than in the low-risk group for both the CGGA and TCGA databases (Figure 5I and J).

### Risk model and macrophage correlation analysis

**Figure 6:**
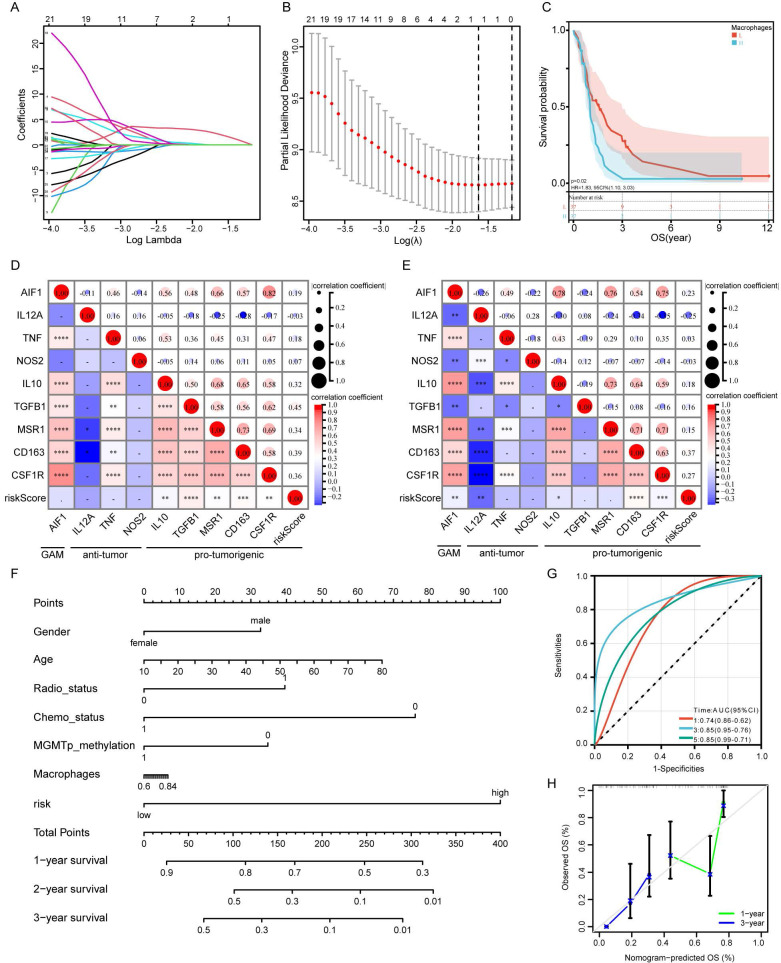
** Correlation analysis of risk score and macrophage.** A: Cross-validation for tuning the coefficient selection in the LASSO regression. B: LASSO regression of the immune-related cells. C: Kaplan-Meier curves display the diversity in OS between the high- and low-infiltration of macrophage in CGGA_325 database. D-E: The correlation between risk score and glioma-associated microglia/macrophages markers in CGGA (D) and TCGA (E) databases. F: Construction of a nomogram for survival prediction. G: AUC of time-dependent ROC curves of the nomogram. H: The calibration curve of the Nomogram.

The results for cell infiltration obtained using the CGGA database were analyzed by LASSO regression, which suggested that macrophages are the most important prognosis associated immune cells (Figure 6A and B). That is, patients with more macrophage infiltration had poorer prognosis than those without macrophage infiltration (Figure 6C). We selected specific markers to represent the polarization of macrophages in GBM. Risk score was positively correlated with M2 macrophage markers, but not with M1 macrophage markers (Figure 6D and E). These results suggest that high risk scores are associated with enrichment of M2 macrophages in glioma, which indicates a pro-tumorigenic phenotype. A nomogram was constructed based on macrophage infiltration, risk score, gender, age, radiotherapy status, chemotherapy status, and MGMT promoter status to predict the prognosis of GBM patients (Figure 6F). This nomogram was used to predict the prognosis of patients with GBM and was found to show high accuracy and stability for prediction of 1-year, 3-year and 5-year prognosis (Figure 6G). The calibration curve of the Nomogram is shown in Figure 6H.

### Risk model and immune checkpoint correlation analysis

**Figure 7:**
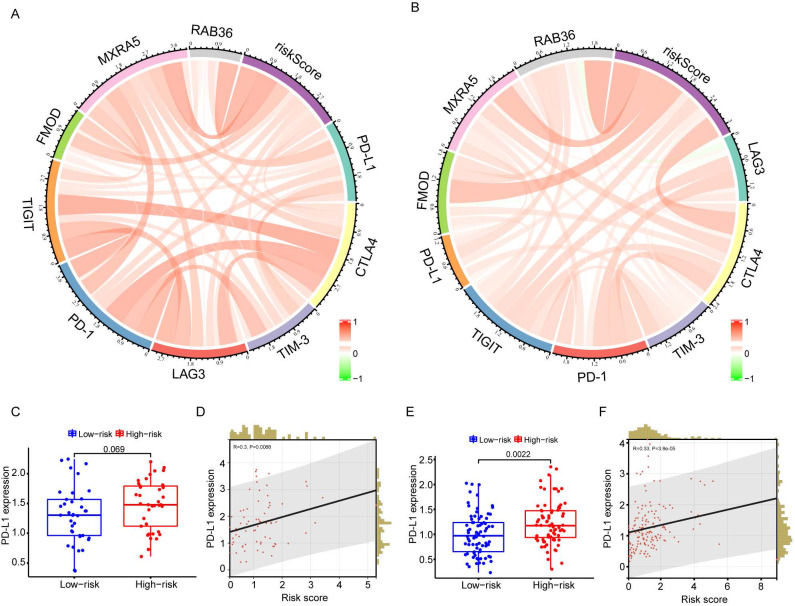
** Correlation analysis of risk score and immune checkpoint genes.** A-B: The correlation between risk score and immune checkpoints in CGGA (G) and TCGA (H) databases. C: The difference of PD-L1 gene expression between high- and low-risk groups in CGGA database. D: Correlation analysis of risk score and PD-L1 expression in CGGA database. E: The difference of PD-L1 gene expression between high- and low-risk groups in TCGA database. F: Correlation analysis of risk score and PD-L1 expression in TCGA database.

To test whether this predictive model could predict immunotherapy responses in GBM patients, a number of immune checkpoints that have been identified as therapeutic targets in glioma by clinical or preclinical trials were included in the analysis. These included PD-1, PD-L1, CTLA-4, TIM-3, TIGIT, and LAG3. Circos plots showed that risk score was strongly associated with immune checkpoint genes both in the CGGA (Figure 7A) and TCGA databases (Figure 7B). PD-L1 has been reported to be upregulated in high-grade gliomas compared with low-grade gliomas. Correlation analysis between risk score and PD-L1 expression showed that PD-L1 was highly expressed in high-risk patients (Figure 7C-F).

**Figure 8:**
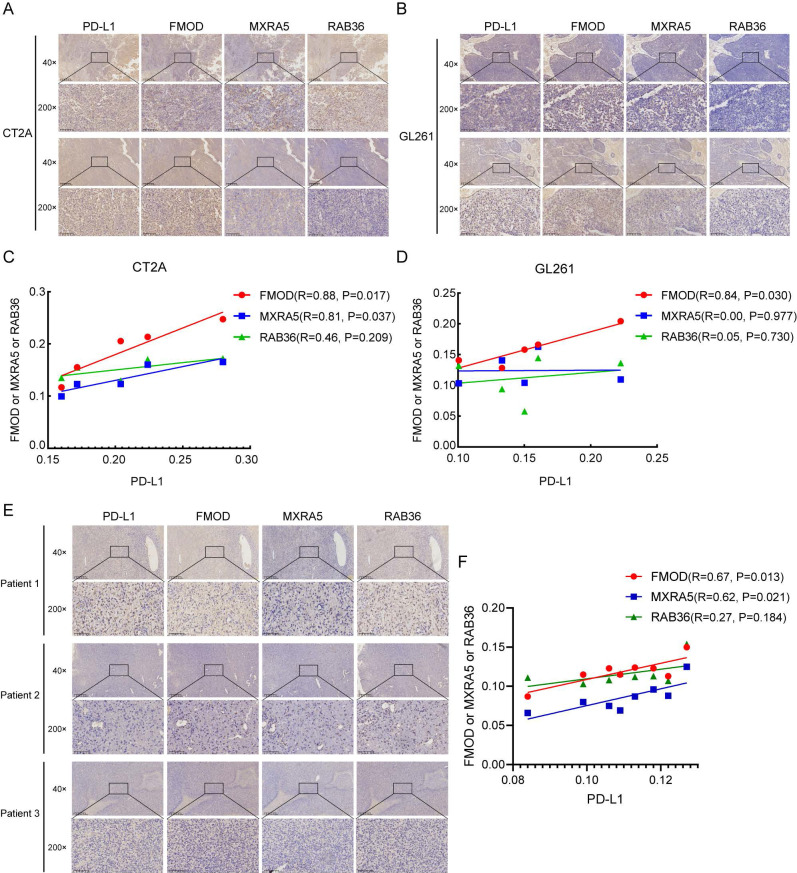
** The IHC analysis of PD-L1, FMOD, MXRA5 and RAB36 in mouse model and GBM patients.** A-B: The IHC of PD-L1, FMOD, MXRA5 and RAB36 in the same mouse tissue in the CT2A (A) and GL261 (B) groups. C-D: Correlation analysis of IHC results of PD-L1, FMOD, MXRA5 and RAB36 in the CT2A (C) and GL261 (D) groups. E: The IHC of PD-L1, FMOD, MXRA5 and RAB36 in three GBM patients. F: Correlation analysis of IHC results of PD-L1, FMOD, MXRA5 and RAB36 in eight GBM patients.

To further verify the correlations of PD-L1 with FMOD, MXRA5 and RAB36, we established a mouse model using CT2A and GL261 mouse glioma cells. The *in vivo* imaging results of mice are shown in Supplementary Figure 1E. Figure 8A and B shows IHC results for PD-L1, FMOD, MXRA5, and RAB36 in the same mouse tissue for the CT2A and GL261 groups. Correlation analysis showed that in the CT2A group, expression of FMOD and MXRA5 was positively correlated with that of PD-L1 (Figures 8C, FMOD with PD-L1, R=0.88, P=0.017; MXRA5 with PD-L1, R=0.81, P=0.037). In the GL261 group, only FMOD was positively correlated with PD-L1 (Figures 8D, FMOD with PD-L1, R=0.84, P=0.030).

Furthermore, we conducted immunohistochemical experiments to examine the protein expression levels of FMOD, MXRA5, and RAB36 in tissue samples from 8 patients with IDH-wildtype GBM. The basic information of the eight patients is provided in Supplementary File 1. Simultaneously, we also detected the expression of PD-L1 and performed a correlation analysis. The results indicated a positive correlation between FMOD and MXRA5 expressions with PD-L1 expression (Figures 8E-F, FMOD with PD-L1, R=0.67, P=0.013; MXRA5 with PD-L1, R=0.62, P=0.021). In contrast, no statistically significant correlation was found between RAB36 expression and PD-L1 expression (RAB36 with PD-L1, R=0.27, P=0.184). Thus, the results suggest that PD-L1 may be a potential immunotherapy target in patients with high-risk scores.

## Discussion

IDH-wildtype GBM is a common intracranial tumor with a high degree of malignancy, characterized by rapid growth, strong invasiveness, and poor prognosis^12^. Therefore, there is an urgent need to find clinical prognostic factors and immunotherapy targets for GBM. Owing to the rapid development of sequencing technology, research has increasingly used sequencing databases to find information related to prognosis of GBM patients.

Researchers utilizing the information from the CGGA database have employed the ESTIMATE algorithm to evaluate the stromal and immune scores of ATRX-mutated versus wild-type (ATRX-wt) glioma tissues. Through LASSO and Cox regression analyses, they have identified seven crucial prognostic genes (HOXA5, PTPN2, WT1, HOXD10, POSTN, ADAMDEC1 and MYBPH). Their risk-model holds potential for diagnostic applications, prognosis prediction, and therapeutic planning in ATRX-wt glioma patients^13^. Furthermore, researchers have conducted differential expression analysis of pyroptosis-related regulators in gliomas, leading to the development of a risk model based on pyroptosis-associated genes. This model sheds light on the relationship between pyroptosis and glioma prognosis^14^.

However, many current studies rely solely on a single database to establish prognostic risk models, potentially leading to biased and unreliable results. In our study, we have overcome this limitation by constructing a GBM prognostic model based on RNA sequencing data from both TCGA and CGGA patients with GBM. Within the TCGA database, we identified 1,274 DEGs associated with prognosis. Similarly, 658 prognosis-related DEGs were determined in the CGGA database. By intersecting these two sets of DEGs, we pinpointed 106 commonly occurring DEGs, suggesting they are likely core genes intimately related to the prognosis of IDH-wildtype GBM patients. Further analysis of these 106 genes reinforced the reliability of a prognostic risk model comprising FMOD, MXRA5, and RAB36. Notably, in both TCGA and the GES43378 validation dataset, patients with high-risk scores exhibited significantly lower overall survival (OS) compared to those with low-risk scores (Figure 3), demonstrating the effectiveness of our model in predicting the prognosis of GBM patients.

FMOD is a proteoglycan, an important component of ECM^15^. It promotes ECM remodeling, which has important roles in tissue repair, tumor formation, and progression^15^. The FMOD gene has been shown to be expressed more frequently in highly malignant GBM than in the relatively benign pilocytic astrocytoma^16^. In addition, Mondal et al. found that FMOD was overexpressed in GBM owing to loss of promoter methylation and showed that the release of FMOD could induce glioma cell migration by promoting the formation of filamentous actin stress fibers^17^. Sengupta et al demonstrated that FMOD promoted glioma growth through angiogenesis induced by endothelial integrin-dependent Notch signaling^18^. Thus, FMOD has the potential to serve as a biomarker and at least in brain tumors, as a marker of disease severity. Through analysis of GBM sequencing data, we also determined that FMOD has an important role in prognosis of patients with GBM, consistent with the conclusions of other researchers. Pending further studies, FMOD could have potential clinical applications as a biomarker.

MXRA5 is a secretory glycoprotein that participates in cell adhesion and ECM remodeling and is associated with tumorigenesis^19^. Somatic mutations in MXRA5 have been observed in non-small-cell lung carcinoma patients^20^. In addition, it is highly expressed in colorectal cancer and can be used as a biomarker for early diagnosis^21^. MXRA5 expression is abnormally high in gliomas and increased with grade, with higher expression in GBM than in lower-grade gliomas^22^. It has also been shown that MXRA5 is closely related to the immune microenvironment of gliomas, especially the infiltration of M2 macrophages^22^. In the present study, M2 macrophage infiltration was increased in patients with high-risk score, possibly owing to expression of MXRA5. These results support the potential value of MXRA5 as a biomarker and target for immunotherapy.

RAB36 is a member of the RAS oncogene family^23^. There have been few studies of its role in tumorigenesis; however, results suggest that it may be related to development and metastasis of bladder cancer^24^. RAB36 has also been reported to be involved in the mechanism of liver metastasis of colon cancer^25^. Thus, FMOD, MXRA5, and RAB36 genes all have important roles in the development of tumors. According to our analysis of the CGGA, TCGA, and GSE43378 datasets, GBM patients with high-risk scores had significantly shorter OS than those with low-risk scores. The roles of these three genes in the tumorigenesis and development of GBM are worth further exploration.

KEGG and GO analyses showed that pathways associated with high-risk scores were enriched with respect to immune-related responses (Figure 5). These results suggest that FMOD, MXRA5, and RAB36 could play important parts in immune response. Immune cells are key components of TME that regulate the progression and recurrence of gliomas. Specifically, owing to the immune escape mechanism, tumor-associated immune cells can promote the development of tumors^26^. In addition, tumor cells can regulate the phenotype and function of immune cells by secreting cytokines and chemokines, causing immune cells to develop in a direction conducive to tumor growth, thereby forming a suitable microenvironment for tumor progression^27^. Our ssGSEA results showed that immune cell infiltration in the high-risk group was generally higher than that in the low-risk group. This suggests that an immunosuppressive microenvironment conducive to tumor growth and evolution is formed in high-risk GBM patients through tumor-immune cell interactions, owing to excessive immune cell infiltration. In addition, studies have shown that immune cells around a tumor can form immune barriers in the tumor marginal microenvironment; this may also promote the recurrence and malignant progression of gliomas and the development of resistance to radiotherapy and chemotherapy^28^.

Through further analysis of the ssGSEA results, we found that macrophages were closely related to the prognosis of GBM patients, with patients with high macrophage infiltration having significantly lower OS compared with those with low macrophage infiltration (Figure 6C). Zhang et al. analyzed sequencing data from 1619 glioma patients and established a prognostic risk score model^29^. They also found that high-risk patients had higher immune cell complexity, and there was a significant positive correlation between risk score and macrophage abundance^29^. Through immunohistochemical experiments, they confirmed that macrophage enrichment in the high-risk group promoted tumorigenesis rather than an anti-tumor state. Their results further support our conclusion that macrophage enrichment in gliomas is likely to contribute to tumor development. Tumor-associated macrophages (TAMs) are an important type of glioma-infiltrating immune cell, accounting for 30-40% of cellular components in GBM^30^. TAM-targeted immunotherapy could thus be a promising method for treatment of GBM^31^. Owing to their angiogenic and immunosuppressive effects, TAMs are generally considered to be promoters of tumor proliferation^32^. M0 macrophages can be polarized to M1 or M2 phenotypes by signaling in the TME^33^. M1 macrophages can produce proinflammatory cytokines and have a tumor-killing role in GBM^32^, whereas, M2 macrophages are largely involved in the promotion of tumor cell proliferation and are associated with poor clinical outcomes in GBM patients^34^. Therefore, we classified infiltrating macrophages in GBM and found that high-risk scores were strongly associated with M2 macrophage markers (Figure 6D, E). There was no correlation between risk scores and GAM markers or M1 macrophage markers. Hussain et al. isolated and analyzed the immune functions of macrophages from postoperative tissue samples of glioma patients, and found that macrophages were more likely to polarize to immunosuppressive M2 phenotype^35^. Andersen et al also found that high expression of M2 macrophage markers CD204 and CD163 in GBM predicted poor prognosis and invasive phenotypes of gliomas^36^. These results suggest that the M2 macrophage subtype may play a major part in GBM. In addition, we established a prognostic nomogram that combines macrophage infiltration with our risk model to more accurately predict outcomes in GBM patients (Figure 6F, G).

In GBM, response to Immune checkpoint inhibitors (ICIs) treatment varies from patient to patient, owing to inherent tumor heterogeneity, with the treatment proving ineffective in most patients. None of the large phase III studies of PD-1 inhibitors for GBM showed any survival benefit^37^,^38^, and fewer than 10% of patients with recurrent GBM respond to treatment with PD-1 inhibitors^39^. Therefore, understanding the molecular mechanisms of immune cell infiltration and immune escape in GBM to improve the efficacy of ICI therapy is a current research challenge^40^. In many types of cancer, the PD-L1 protein level is considered to be a key predictive marker of response to PD-1/PD-L1 antibody treatment^39^. PD-L1 is frequently expressed in all grades of gliomas and is positively correlated with WHO grade^40^. There currently is no feasible protocol to determine which patients will benefit from ICI treatment. Our preliminary analysis results show significant differences in PD-L1 gene expression between the high-risk and low-risk groups. Our TCGA and CGGA database analyses showed that PD-L1 was up-regulated in high-risk GBM patients (Figure 7C, E), and risk score was positively correlated with PD-L1 expression (Figure 7D, F). This suggests that anti-PD-L1 treatment could be a potential effective immunotherapy for patients with high-risk scores. Therefore, our risk model may help more accurately identify GBM patients who respond to PD-L1 immunotherapy and prolong their survival. However, our three-gene signature model was analyzed from databases, there is still a lot of work to be done before it can be truly applied to clinical patients.

The expression of PD-L1 has also been reported to be related to infiltration of M2 macrophages and neutrophils, with high expression of PD-L1 positively correlated with M2 polarization^41^. Our results confirmed this link; that is, M2 macrophage infiltration and PD-L1 expression were increased in patients with high-risk scores. As PD-L1 can promote the polarization of M2 macrophages, targeting PD-L1 may be a promising method to transform macrophages to the anti-tumor M1 phenotype. Indeed, Hartley et al. found that PD-L1 was involved in the formation of the immunosuppressive phenotype of macrophages^42^, and inhibition of PD-L1 can activate macrophages and promote their proliferation and survival. A study of the effects of ICI treatment in a mouse sarcoma model also demonstrated immune-activated remodeling of intra-tumoral macrophages^43^, whereas in preclinical study, a combination of PD-L1 and PD-1 antibodies was effective in the treatment of GBM patients^44^-^46^. Thus, our study and those of others have shown that PD-L1 antibodies may produce additional anticancer effects that promote the polarization of macrophages.

## Conclusion

In conclusion, we have established a three-gene risk model based on analysis of sequencing data of GBM patients. Our risk model can effectively predict the prognosis of patients with GBM, and GSEA showed that it is closely related to tumor immunity. Through analysis of immune cell infiltration, we found that macrophage infiltration is an important factor in the prognosis of GBM patients. Analysis of gene expression at immune checkpoints suggests that PD-L1 is a potential treatment target for patients with high-risk scores.

## Supplementary Material

Supplementary figure, table, files.
