# Supplementary material for: Identification of an Immune signature assisted prognosis, and immunotherapy prediction for IDH wildtype glioblastoma
Source: J Cancer. 2024 Oct 21;15(19):6452–67. doi: 10.7150/jca.100144 (PMC11540507; doi:10.7150/jca.100144)
Supplement: Supplementary file 1 — Supplementary figure, table, files. [file jcav15p6452s1.zip › Supplementary/Supplementary figures and tables.pdf]

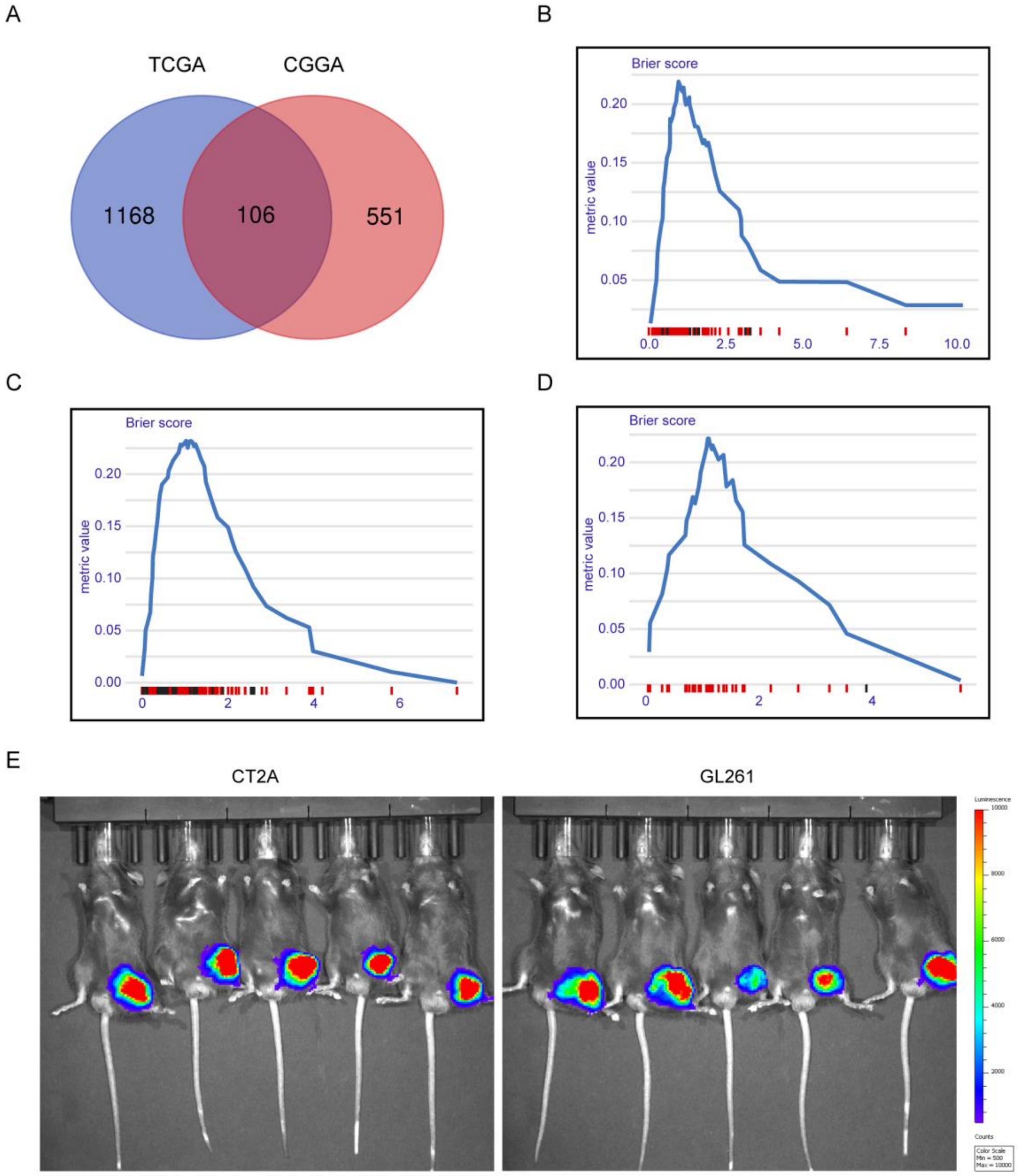

Supplementary Figure 1

A: Venn diagram of differentially expressed genes in TCGA and CGGA databases. B: The Brier score curve of CGGA database. C: The Brier score curve of TCGA database. D: The Brier score curve of GSE43378 datasets. E: In vivo imaging images of mouse models established using mouse glioma cells CT2A and GL261.

Supplementary Table 1 Baseline data table for WHO Grade 4 glioma patients in database.

	TCGA(N=155)	CGGA(N=74)	GSE43378(N=36)
Characteristics			
Grade			
WHO Grade 4	155(100%)	74(100%)	36(100%)
WHO Grade 3	0(0%)	0(0%)	0(0%)
WHO Grade 2	0(0%)	0(0%)	0(0%)
PRS_type			
Primary	155(100%)	74(100%)	36(100%)
Recurrent	0(0%)	0(0%)	0(0%)
Secondary	0(0%)	0(0%)	0(0%)
Age(years old)			
≥60	82(52.9%)	17(23%)	15(41.7%)
<60	73(47.1%)	57(77.0%)	21(58.3%)
Gender			
Female	54(34.8%)	26(35.1%)	12(33.3%)
Male	101(65.2%)	48(64.9%)	24(66.7%)
IDH_mutation			
Mutant	9(5.8%)	0(0%)	0(0%)
Wildtype	140(90.3%)	74(100%)	0(0%)
NA	6(3.9%)	0(0%)	36(100%)
MGMTp_methylation_status			
methylated	0(0%)	23(31.1%)	0(0%)
un-methylated	0(0%)	49(66.2%)	0(0%)
NA	155(100%)	2(2.7%)	36(100%)
